# An Incidental Discovery of Rectal Leiomyoma in an Asymptomatic Patient: A Case Report

**DOI:** 10.7759/cureus.70512

**Published:** 2024-09-30

**Authors:** Shahd Yaghi, Murad Qirem, Muhammad Hussain, FNU Marium, Scott W Digiacomo

**Affiliations:** 1 Internal Medicine, Saint Michael's Medical Center, Newark, USA; 2 Gastroenterology and Hepatology, Saint Michael's Medical Center/New York Medical Center, Newark, USA; 3 Internal Medicine, Jinnah Sindh Medical University, Karachi, PAK; 4 Gastroenterology and Hepatology, Saint Michael's Medical Center, Newark, USA

**Keywords:** colonoscopy, fibroid, leiomyoma, rectal, rectal leiomyoma

## Abstract

Leiomyomas are benign smooth muscle tumors predominantly found in the uterine wall, with rare occurrences in the gastrointestinal tract. Here, we report a rare case of a leiomyoma that was incidentally found in the rectum of an asymptomatic male who underwent a routine screening colonoscopy. Histopathological examination confirmed the diagnosis of leiomyoma. This case emphasizes the significance of routine colonoscopic screenings in the early detection of asymptomatic colorectal lesions and the pivotal role of Immunohistochemistry in differentiating rectal leiomyomas from other mesenchymal neoplasms, guiding appropriate management strategies.

## Introduction

Leiomyomas are benign smooth muscle tumors that can arise in the muscularis mucosae or the muscularis propria. Lesions in the muscularis propria usually require surgical removal (if deemed necessary) while lesions in the muscularis mucosase can be removed endoscopically. They occur commonly in the uterine wall. However, it can arise from the smooth muscle tissue anywhere in the body, including, to a lesser extent, the gastrointestinal tract. Rectal leiomyomas, however, are extremely rare; thus, their diagnosis and management pose quite a challenge to the physician [1,2].

The causes of rectal leiomyomas are not well known, although, usually, the etiology lies in genetic factors or abnormalities in the development of smooth muscle cells. The tumor is benign, composed of spindle-shaped smooth muscle cells, and bounded by connective tissue [1].

In the case below, we report a rare occurrence of leiomyoma in the rectum, and we highlight the importance of immunohistochemical stains in the diagnosis.

## Case presentation

**Figure 1:**
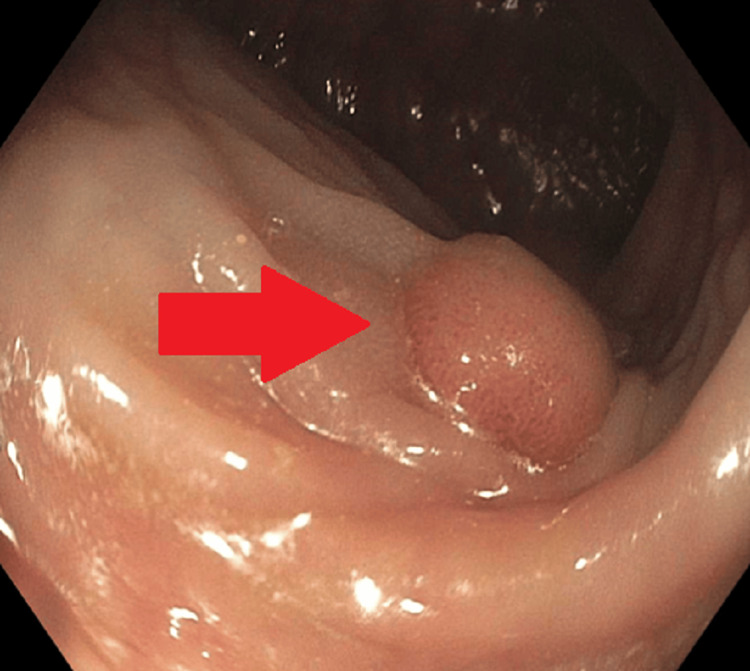
Colonoscopic picture of the leiomyoma in the rectum Arrow points to the leiomyoma

A 66-year-old gentleman with no remarkable medical history visited the clinic to undergo a first-time screening colonoscopy. During the visit, the patient denied any gastrointestinal symptoms. However, despite being asymptomatic, a fairly distinct 8 mm sessile polyp with a regular pit pattern was seen in the rectosigmoid colon on the colonoscopy (Figure 1), which was removed using a hot snare technique and then sent for pathology.

**Figure 2:**
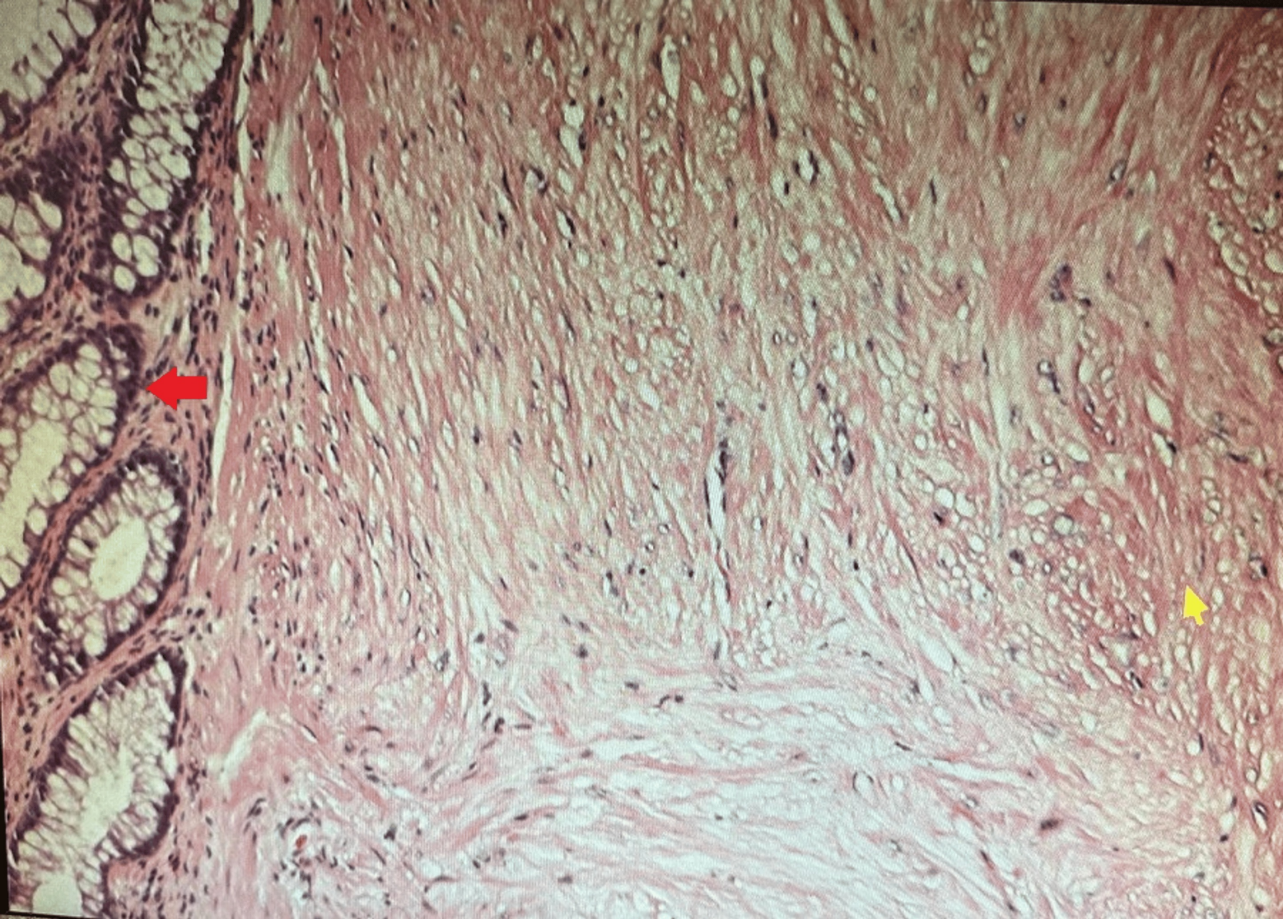
Rectal leiomyomas on histology The yellow arrow points to the smooth muscle tissue of the leiomyomas. The red arrow points to the normal rectal tissue.

Pathological examination revealed submucosal spindle cell neoplasm with features favoring leiomyoma with clear margins (Figure 2). It confirmed its origin as smooth muscle, with positive immunohistochemistry stains for desmin and smooth muscle actin (SMA). In addition, CD34 was negative. This was consistent with leiomyoma located in the muscularis mucosae and a complete resection was confirmed on histology.

In the absence of any symptoms, this incidental finding proves the importance of routine screening colonoscopies in detecting asymptomatic colorectal lesions. In this patient, since the leiomyoma was completely excised with the hot snare, he will not require more frequent screening and will be regularly followed up for a repeat screening colonoscopy in 10 years.

## Discussion

Symptoms related to leiomyomas located in the rectal area can have a wide range of symptoms, including change in bowel habits, rectal bleeding, and abdominal pain, or it could be completely asymptomatic [1,3]. Given the non-specific character of all these symptoms, a correct diagnosis requires a combination of imaging techniques and histopathological evaluations. Endoscopy, computed tomography (CT), and magnetic resonance imaging (MRI) play critical roles in determining the size, location, and features of rectal leiomyomas. In addition, rectal EUS especially when combined with fine needle biopsy has great diagnostic accuracy in diagnosing leiomyomas [1,4].

Rectal leiomyomas may be difficult to distinguish from other mesenchymal cancers, mainly gastrointestinal stromal tumors (GISTs). Immunohistochemical analysis is helpful in these entities' differentiation. Generally, leiomyomas are quite characteristic in their profiles in immunohistochemistry. Stains targeting smooth muscle-specific markers like SMA and desmin support their derivation from the smooth muscle, while CD34 and CD117 are negative in leiomyoma but positive in GIST [5,6]. Accurate diagnosis will guide the right options for therapy.

Rectal leiomyoma management techniques are impacted by tumor size, location, and clinical presentation [1]. Small, asymptomatic leiomyomas may be appropriate for surveillance due to their benign nature. However, larger or symptomatic tumors frequently necessitate intervention, with surgical excision being a typical method [1]. The NCCN Task Force report underlines the necessity of developing tailored treatment programs that consider patient characteristics and potential risks [7].

Rectal leiomyomas, which are benign in origin, generally have a good prognosis. Patients frequently have symptom relief after surgical excision, with a low chance of recurrence. Long-term follow-up is required to watch for potential problems or recurrence, albeit such cases are uncommon in the literature [7].

## Conclusions

Rectal leiomyomas, although benign, can significantly impact patient well-being. Accurate diagnosis through a combination of imaging and histopathological assessment is paramount. The choice of management, whether surveillance or surgical intervention, should be individualized based on tumor characteristics and patient factors. Continued research and collaborative efforts are essential for refining diagnostic and therapeutic approaches, ultimately improving outcomes for individuals with rectal leiomyomas.

## References

[1] DeMatteo RP, Lewis JJ, Leung D, Mudan SS, Woodruff JM, Brennan MF (2000). Two hundred gastrointestinal stromal tumors: recurrence patterns and prognostic factors for survival. Ann Surg.

[2] Fasih N, Prasad Shanbhogue AK, Macdonald DB (2008). Leiomyomas beyond the uterus: unusual locations, rare manifestations. Radiographics.

[3] De Palma GD, Rega M, Masone S (2009). Lower gastrointestinal bleeding secondary to a rectal leiomyoma. World J Gastroenterol.

[4] Silvia RDP, Saad-Hoossne R, Ferraz RA (2011). Treatment of rectal leiomyoma by endoscopic resection. J Coloproctol.

[5] Osborn M, Weber K (1982). Immunofluorescence and immunocytochemical procedures with affinity-purified antibodies: tubulin-containing structures. Methods Cell Biol.

[6] Miettinen M, Sarlomo-Rikala M, Lasota J (1999). Gastrointestinal stromal tumors: recent advances in understanding of their biology. Hum Pathol.

[7] Demetri GD, von Mehren M, Antonescu CR (2010). NCCN Task Force report: update on the management of patients with gastrointestinal stromal tumors. J Natl Compr Canc Netw.

